# Remote sensing-derived time series of transient glacier snowline altitudes for High Mountain Asia, 1985–2021

**DOI:** 10.1038/s41597-024-04309-6

**Published:** 2025-01-17

**Authors:** David Loibl, Niklas Richter, Inge Grünberg

**Affiliations:** 1 Geography Department, Humboldt-Universität zu Berlin, Unter den Linden 6, 10099 Berlin, Germany; 2 Department of Atmospheric and Cryospheric Sciences, Universität Innsbruck, Innrain 52, 6020 Innsbruck, Austria; 3 Division: Geosciences | Permafrost Research, Alfred Wegener Institute - Helmholtz Center for Polar and Marine Research, Telegrafenberg A45, 14473 Potsdam, Germany

**Keywords:** Cryospheric science, Environmental health

## Abstract

This study presents a new dataset of remote sensing-derived Transient Snowline Altitude (TSLA) measurements for glaciers in High Mountain Asia. We use the Google Earth Engine to obtain TSLA data for approx. 2.8 · 10^4^ glaciers larger than 0.5 km^2^. After filtering and postprocessing, the dataset comprises ca. 9.66 million TSLA measurements with an average of 341 ± 160 measurements per glacier, covering the time span 1985 to 2021.

## Background & Summary

A key challenge in glaciology is obtaining time series of glacier change that cover multiple decades at high temporal resolution^1^. Such data series are essential for analyzing glacier development and its drivers over large regions. Only two out of the ~95,000 glaciers in High Mountain Asia (HMA) have a continuous time series of mass balance measurements covering more than 30 years (World Glacier Monitoring Service’s ‘reference glaciers’). Considering that recent changes of glaciers in HMA are known to be heterogeneous in space and time^2–4^, there is a substantial knowledge gap regarding the actual dynamics at individual glaciers and their forcing.

To date, observational glaciological assessments on regional-to-continental scales rely either on remote sensing-based measurements of geodetic mass balances^2,5^ or gravity anomalies^6–8^. These datasets have strongly improved our knowledge on large-scale patterns of glacier change in HMA. However, they do not resolve intra-annual variations. The integrative approaches behind these data thus limit their potential towards analyses of specific drivers and respective sensitivities. This knowledge gap in temporal detail of observations hinders analyses of current and predictions of future glacier variability.

Hence, there is a need for an observational glaciological dataset that (i) resolves the annual/seasonal variability of individual glaciers, (ii) spans multiple decades to infer influences of climatic changes, and (iii) covers the entire region of HMA to unravel spatial patterns of glacier change.

The Transient Snowline Altitude (TSLA) on a glacier surface integrates topographic and climatic conditions which are affecting a glacier’s accumulation and melt regimes at a specific point in time^9,10^. The maximum TSLA during the ablation season corresponds to the Equilibrium Line Altitude (ELA) of a glacier, which in turn is indicative of the overall mass balance of a glacier^11^. Due to the marked contrast between the optical properties of snow-covered and snow-free parts of a glacier surface, TSLAs of individual glaciers can be estimated at high spatial resolution from multispectral satellite imagery, which have been available since the mid 1980s, e.g. provided by the Landsat program. Given the high spatial resolution and long temporal span of the source data, TSLA data bears considerable potential towards closing the scale gaps in observational data of glacier change and variability^12,13^.

The prospects of TSLA data regarding the analysis of individual glaciers have been demonstrated in case studies on local to regional scales. Examples include applications of TSLAs to calibrate glaciological models^14^, to infer seasonally to annually resolved mass balance time series^15^, and to disentangle topographic and climatic drivers of glacier change^16^.

Here, we present a novel dataset of TSLA measurements covering all glaciers >0.5 km^2^ in HMA (n = 28,336 based on Randolph Glacier Inventory (RGI) version 6 data) for a time frame from 1985 to late 2021 based on more than 10^5^ Landsat satellite images^17^. Our dataset allows for investigations of the characteristics of glacier change at unprecedented spatio-temporal resolution and coverage. The TSLA time series may be used to calibrate glaciological models and for regression-based analyses to infer meteorological drivers of glacier change. It therefore has great potential to contribute to filling the gap of lacking observational data in remote high mountain environments.

We created the ‘MountAiN glacier Transient snowline Retrieval Algorithm’ (MANTRA)^18^, a band ratio-based Google Earth Engine (GEE)^19^ routine, and used it to obtain ca. 24.5 million TSLA measurements from 104,155 unique Landsat 4, 5, 7 and 8 scenes. After threshold-based filtering for situations with adverse sensing conditions, e.g. too much cloud cover or too few well-illuminated surfaces on a particular glacier, approx. 9.66 million TSLA measurements from 100,012 unique Landsat scenes remained.

## Methods

### Input data

#### Satellite imagery

Satellite imagery from the Landsat 4^20^, 5^21^, 7^22^ and 8^23^ sensors^24^ provided the multispectral base data for the classification of surface material. To ensure data quality and reproducibility, we used calibrated top-of-atmosphere^25^ Tier 1 products for the classification of surface materials. We configured the algorithm to preselect scenes for glacierized regions of HMA with less than 80% total cloud cover. For each scene, the respective sensor’s bands green (G), near infrared (NIR), both short wave infrared (SWIR1, SWIR2) and thermal (T) were extracted. Since the subsequent band ratio approach includes thermal infrared, other multispectral sensors lacking a thermal channel, such as Sentinel-2, could not be considered.

The vast majority of scenes used for processing were obtained by Landsat 5 (37,739), Landsat 7 (32,830), and Landsat 8 (29,231), while only 212 scenes originate from Landsat 4. Since the TSLA processing algorithm does not perform any preselection except for ruling out scenes with more than 80% total cloud cover, this uneven distribution is directly related to the availability of Landsat scenes in general and specifically in GEE.

#### Glacier outlines

Glacier outlines were obtained from the RGI version 6^26^. We chose to use RGI6 as the single source of outline data to ensure a consistent basis for classification throughout the time series. The effect of glacier area change was considered to be of subordinate importance in this regard, because the elevation metric TSLA is hardly sensitive to changes in area.

#### Digital elevation model

We chose the ALOS World 3D 30 m resolution (AW3D30)^27^ digital elevation model (DEM) as the basis for altitude measurements and geomorphometric calculations owing to its expedient data quality for HMA^28–31^.Applying the same reasoning as with the glacier outlines, we prioritized a single integrating DEM dataset to ensure consistency throughout the time series over attempting to reflect changes in surface elevation by using multiple DEMs.

### Transient snowline altitude data retrieval

We applied GEE to obtain TSLA measurements. Initial tests showed that deficiencies in glacier outline delineation and low pixel count adversely affect the classification accuracy with smaller glaciers. Based on these empirical findings, we omitted all glaciers smaller than a threshold value of 0.5 km^2^.

**Fig. 1:**
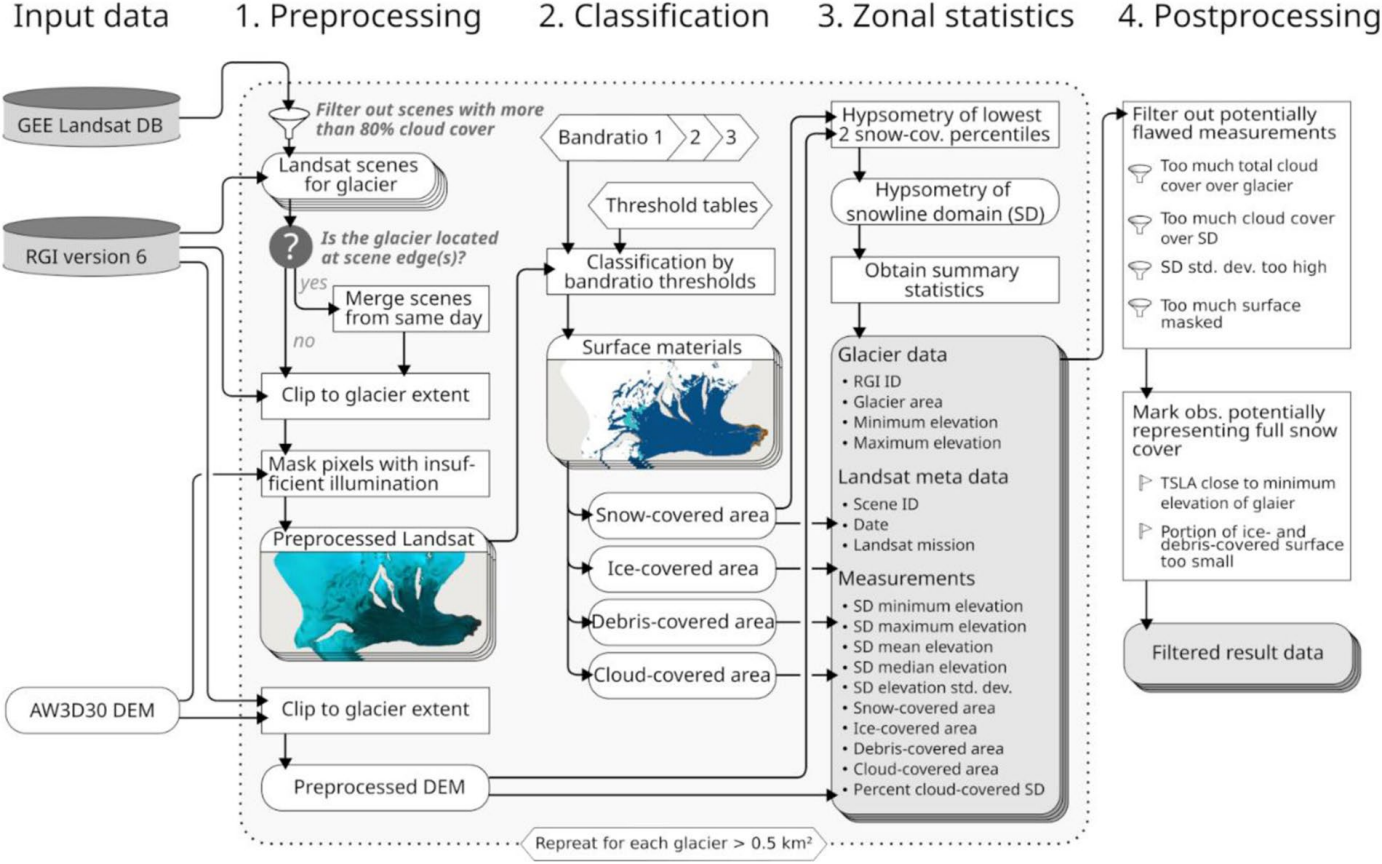
Processing scheme of the Transient Snowline Altitude (TSLA) retrieval and filtering algorithm. Preprocessing, classification, and zonal statistics were performed in Google Earth Engine (GEE). The postprocessing was conducted in Python.

For each glacier, the algorithm firstly checks for available Landsat scenes and clips them to glacier extent (Fig. 1). If a glacier is split between multiple scenes the relevant data is merged. Insufficiently illuminated surfaces are masked using DEM-based topography and sun position during the time of the Landsat data take.

The classification of surface materials aims at distinguishing snow, ice, and debris and is based on a combination of three band ratios: The Normalized Difference Snow Index (NDSI)^32,33^ (G – SWIR1)/(G + SWIR1) and two empirically derived indices (T − G + NIR)/(T + G + NIR) and (G − T)/(G + T). To avoid misclassifications under cold conditions, thermal infrared (T) values below 263.15 K were changed to 263.15 K in the classification. Threshold values for each ratio were adjusted for each Landsat sensor’s radiometric characteristics in an expert-driven manual calibration procedure. The automatic classification was then conducted by testing individual pixels against empirically derived minimum and maximum threshold values for the three ratios. Pixels that did not match any class were classified as cloud cover.

For each glacier and time step, we obtained the distribution of DEM values for the snow-covered area resulting from the previous step. The TSLA was calculated as the median of the elevation band comprised between the minimum and the second percentile. For quality and uncertainty assessments, we additionally calculated minimum, maximum and mean values as well as standard deviation for the same elevation band. Total area and relative portion of each surface material class and cloud cover were also included in the output for each measurement.

**Fig. 2:**
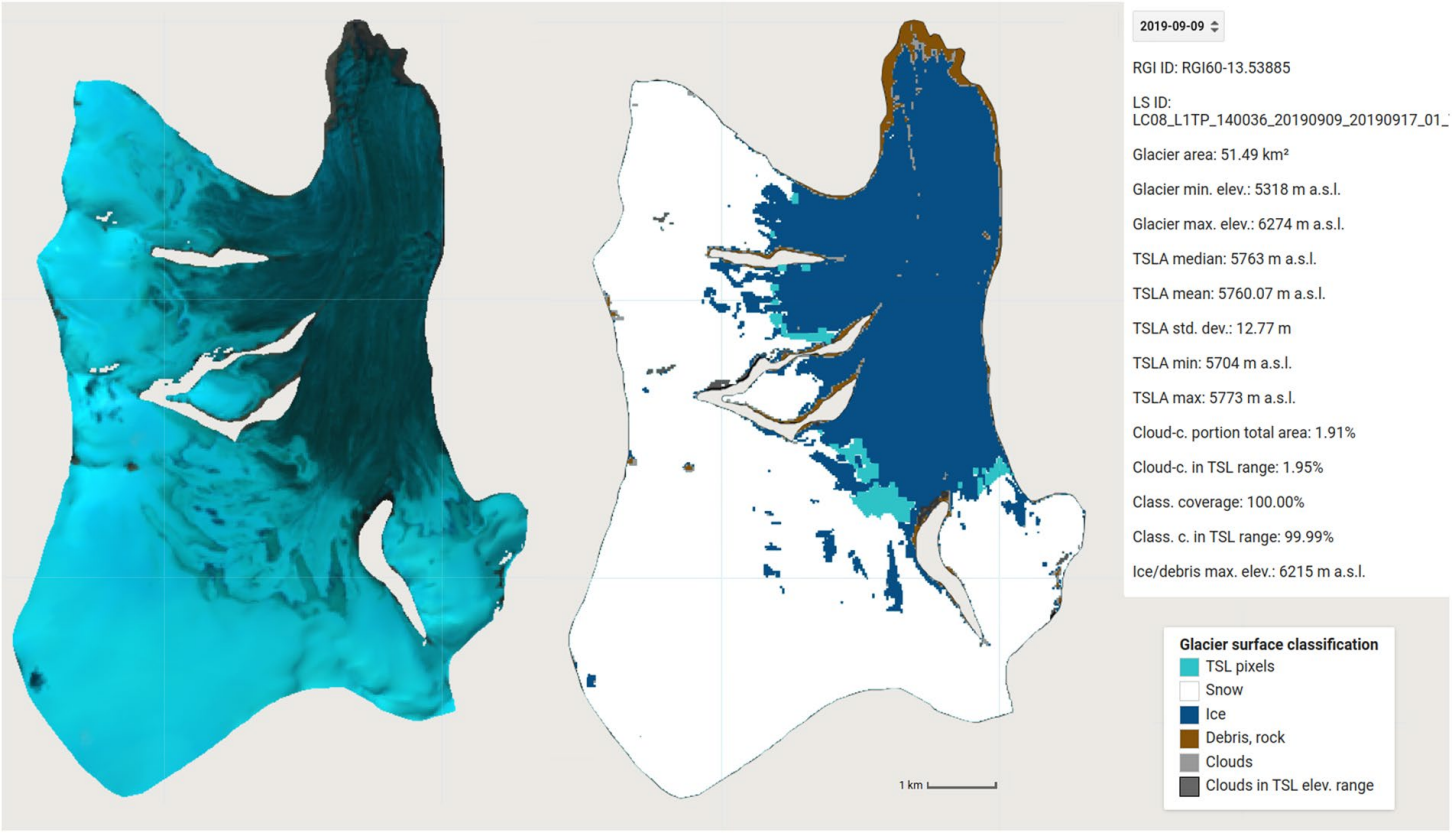
Elements of the MANTRA^18^ visualization tool in Google Earth Engine^19^ (Loibl, 2022), including a false-color image of the satellite scene (left, here Landsat 8^23^ image in bands 6-5-4), a visualization of the classification results (center), and an information box displaying key metadata, result metrics, as well as the date selection menu. Cyan region (‘TSL pixels’) indicates the image part that is used to obtain the TSLA, i.e. the lowest two percentiles of the snow-covered surface.

The MANTRA main routine is optimized for maximum performance in processing comprehensive TSLA datasets and therefore does not include any visualization of the results. To facilitate calibration of the band ratio thresholds and manual inspection of the results, we developed the MANTRA Evaluation Tool, a GEE script that visualizes satellite images and classification results as well as core result metrics (Fig. 2). Based on the same core metrics and threshold as the main data retrieval routine, the tool provides a visualization of the results for a specific glacier and Landsat scene, including a false-color representation of the Landsat image, the classification results, and key result metrics. This tool can be applied at the user’s convenience in the Google Earth Engine. For n = 7 glaciers spanning a wide range of locations across HMA, the full time series were manually evaluated using the visualization tool (see Technical Validation).

### Transient snowline altitude data postprocessing

GEE processing results were subsequently filtered using the following criteria to identify and omit flawed measurements:

- Total portion of unclassified/cloud cover pixels over the whole glacier must be less than 70%,
- Total portion of unclassified/cloud cover pixels in the region used for TSLA measurement (lowest two percentiles of snow cover, see above) must be less than 40%,
- More than 40% of the glacier surface must have been considered in the classification (important for insufficiently illuminated scenes and Landsat 7 after the scan line corrector failure),
- The sum of unclassified/cloud cover pixels (see criterion a) and unconsidered pixels (see criterion c) must be less than 60%,
- The standard deviation in the TSLA measurement must not be greater than 0.2 times the glaciers total elevation range (avoid strong scatter which typically indicates that the TSLA measurement region consists of multiple disconnected parts),
- The elevation range of the TSLA measurement region must not be less than 0.005 times the glaciers elevation range (avoid potentially flawed results when the TSLA measurement region consists of very few or an isolated group of pixels),
- The cumulative area of the debris and ice must be greater than 0.01 km^2^ and greater than 2% of the total glacier area, and
- The TSLA must be at least 50 m above the minimum altitude of the glacier.

The latter two criteria aim to avoid TSLA values remaining at the lowest point of the glacier when it is fully snow covered while the actual snowline altitude lies below the glacier’s altitude range.

TSLA values and maxima from the late summer ablation phase are indicators of melt dynamics and the ELA, respectively, and therefore of particular interest in many subsequent analyses. In our analyses, we defined the ‘ablation phase’ as the time span July to October (JASO). July to September were chosen acknowledging the importance of the late summer months for the melt process. The month of October was added to account for TSLA maxima that were not detectable during summer, typically due to monsoonal cloud cover. Our remote sensing-based measurement routine therefore features the detection of ‘frozen maxima’ when cold and dry weather leads to clear sky conditions in early autumn.

During the winter months, low sun angles and little total illumination frequently lead to challenging conditions for TSLA measurements, particularly through topographic shadows. As the spectral characteristics of shaded snow are close to those of ice, a distinction of reasonable robustness was not possible within our classification approach. Therefore, an additional filtering step was included, removing unrealistically high values during the winter months. Outside the ablation phase, we omitted TSLA observations above 20% of the whole glacier’s elevation range.

## Data Records

The dataset is available at Pangaea^17^. The data is provided as a NetCDF^34^ file. A version that was not filtered for unrealistically high values during the winter months is available on FigShare^35^.

For each of the approx. 9.66 million measurements in the dataset, the information listed in Table 1 is stored. Date and geographical coordinates of each measurement are set up as array indices to facilitate direct access and filtering for subsets. The median elevation of the transient snowline elevation range (*TSLrange_median*) was used as metric for the TSLA in subsequent analyses. Notably, such a MANTRA TSLA measurement represents a single median value per Landsat scene and glacier.

**Table 1:** Data structure of the TSLA dataset.

Label	Description	Unit/format	Derived from
LS_DATE	Date of the Landsat data take	YYYY-MM-DD	Landsat metadata
Lat	Latitude (central point of glacier)	Decimal deg.	RGI v6
Lon	Longitude (central point of glacier)	Decimal deg.	RGI v6
LS_ID	Landsat scene identifier		Landsat metadata
LS_SAT	Landsat satellite		Landsat metadata
RGI_ID	Randolph Glacier inventory identifier		RGI v6
glacier_area	Glacier area	km^2^	RGI v6
glacier_DEM_min	Elevation at lowest point of glacier	m a.s.l.	AW3D30 DEM
glacier_DEM_max	Elevation at highest point of glacier	m a.s.l.	AW3D30 DEM
CC_area	Cloud-covered area	km^2^	Surface classification
DC_area	Debris-covered area	km^2^	Surface classification
IC_area	Ice-covered area	km^2^	Surface classification
SC_area	Snow-covered area	km^2^	Surface classification
TSLrange_max	Highest elevation in TSL range	m a.s.l.	AW3D30 DEM, Surface classification
TSLrange_mean	Mean elevation in TSL range	m a.s.l.	AW3D30 DEM, Surface classification
TSLrange_median	Median elevation in TSL range	m a.s.l.	AW3D30 DEM, Surface classification
TSLrange_min	Lowest elevation in TSL range	m a.s.l.	AW3D30 DEM, Surface classification
TSLrange_stdev	Standard deviation of elevation in TSL range	m	AW3D30 DEM, Surface classificatio
TSLA_norm	Median elevation in TSL range, normalized to glacier elevation range		AW3D30 DEM, Surface classification
TSLA_stdev_norm	Standard deviation of elevation in TSL range, normalized to glacier elevation range		AW3D30 DEM, Surface classification
CC_TSLArange_pct	Portion of cloud-covered surface over TSL range	%	Surface classification
class_coverage	Portion of glacier surface covered with classified pixels (excluding e.g. shadows and Landsat7 SLC error)	%	Surface classification
tool_version	Version of the GEE TSLA tool used for processing the data		GEE processing routine

**Fig. 3:**
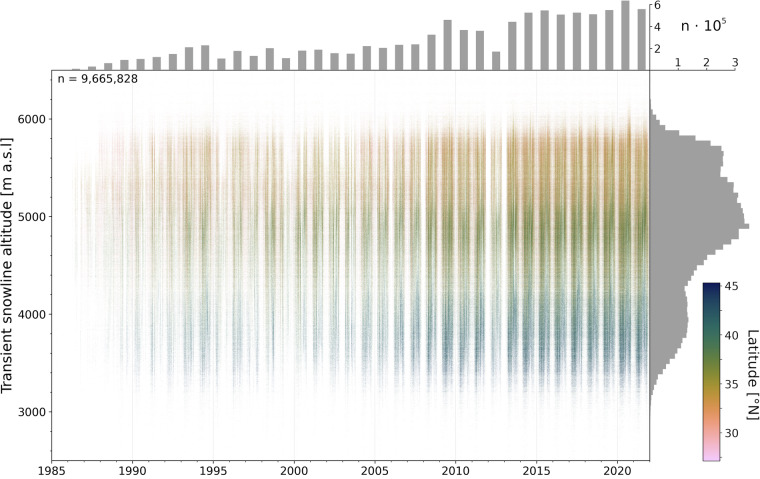
Full time series of TSLA observations after filtering (see Methods Section for details). Color-coded to the latitude of individual glaciers. Gray histograms indicate distribution densities along the x and y axis, that is the number of measurements per year or TSLA band.

The TSLA time series exhibits a distinct sinusoidal base pattern, indicating it is predominantly controlled by the seasonal cycle (Fig. 3). In line with expectations from the northward decreasing gradient of insolation patterns in HMA^36^, TSLA value ranges clearly decrease along a latitudinal gradient. The histogram shows a trimodal distribution, with the overall maximum value count centered around ca. 4900 m a.s.l., and two further maxima at ca. 5550 m a.sl. and ca. 3900 m a.s.l.. Particularly for the lowest mode, it is obvious that the respective TSLAs originate almost exclusively from latitudes poleward of 40°N, i.e., the Tien Shan.

Ninety-nine percent of the measurements are concentrated in an elevation band between 3289 and 5992 m a.s.l.. Nevertheless, individual outliers reach up above 7000 m a.s.l.. Although such extreme TSLAs have been reported^37^, they highlight that individual erroneous data points are inevitable with our highly automated approach despite careful filtering.

**Fig. 4:**
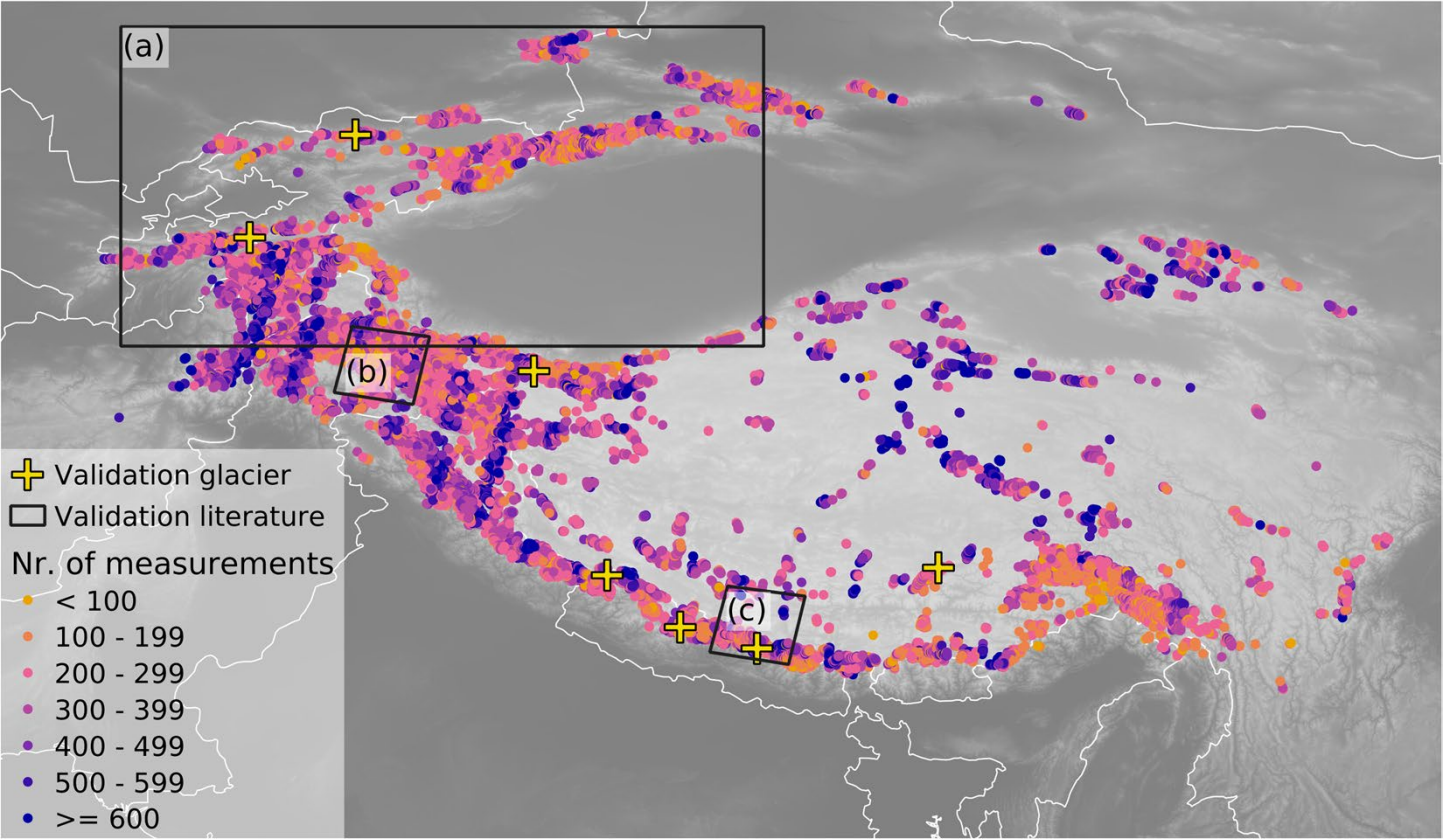
Map of High Mountain Asia including all glaciers colored by the number of TSLA measurements; validation glaciers as used in our study are highlighted with a yellow cross, the black rectangles show areas used for cross-validation with (**a**) Tien Shan and Pamir covered in Barandun *et al*.^3^, (**b**) the Hunza (Karakoram) and (**c**) Langtang (Himalaya), both analyzed in Racoviteanu *et al*.^13^.

Mean and median number of measurements per glacier are ca. 343 ± 156 (σ) and 314 ± 121 (MAD), respectively, for the observation period 1985 to 2021 (Fig. 4). The number of measurements per year generally increases with time from a minimum in 1985 (n = 20) to a maximum in 2020 (n = 632,326; Fig. 3). The most prominent exception to this positive trend is 2012, the year with the fewest measurements (n = 211,494) in the whole decade. As visible in Fig. 3, this minimum in numbers of observations is caused by data gaps in the winters 2011/2012 and 2012/2013, and thus hardly affects TSLA data quality during the ablation season. The differences in the number of observations per year need to be taken into account in further analyses focusing on long-term developments, e.g. trends throughout the time series.

The total number of TSLA measurements per glacier is related to climate conditions such as average cloud cover (Fig. 4). An additional effect is the scan-line error of Landsat 7, which strongly reduced data availability in areas without overlapping stripes (Fig. 4).

## Technical Validation

For each measurement in the TSLA dataset^17^, standard deviation is included as a metric to assess variance and uncertainty. Mean and median standard deviation of the TSL range throughout the entire TSLA dataset are ca. 16.32 m and 11.99 m, respectively.

The quality of the DEM employed to obtain TSLAs is critical to the quality of the resulting measurements. For HMA several studies found that AW3D30 data have fewer and less pronounced errors and an overall better terrain representation than other freely available DEMs, i.e. SRTM and ASTER GDEM^38,39^.

Notably in the context of glaciological analyses, AW3D30 data is photogrammetrically processed from an average of multiple optical images taken by the PRISM sensor between 2006 and 2011^23^, and therefore does not represent the surface elevation at a specific point in time. Also, changes in glacier surface elevations throughout the studied period are not accounted for. Owing to regional differences in mass balance change throughout the last decades, it is impossible to generalize the effects of mass loss or gains. Nevertheless, it can be assumed that deviations increase with temporal difference between DEM and TSL measurement. Considering average glacier elevation ranges of 793 ± 450 m in the MANTRA dataset and −0.27 ± 0.19 m/yr average glacier surface elevation changes (estimate based on data from Azam *et al*.^40^) the effects of surface elevation changes are neglectable in comparison to measurement uncertainty.

### Manual checks

Using the MANTRA Evaluation Tool, we checked the full filtered TSLA time series at seven glaciers (n = 2281 scenes, Fig. 4), representing different key regions of HMA (Table 2). The glaciers were selected either according to spatial distribution and due to relevance in existing glaciological studies. As such, they incorporate, among others, the Abramov Glacier (RGI60-13.18096), the Yala Glacier (RGI60-15.03954), and the Rikha Samba Glacier (RGI60-15.04847). For each scene, we manually assigned the classification result into four categories: (1) TSLA correctly identified, (2) TSLA overestimated, (3) TSLA underestimated and (4) for cases where the classification result was not clearly identifiable. Henceforth, we consider those scenes where the validity could not be confirmed manually (4) as faulty in the accuracy assessment.

**Table 2:** Results of manual checks using the MANTRA Evaluation Tool.

RGI-ID	Season	Correct	Overest.	Underest.	Non-attributable
RGI60-13.11609	Full year	267	47	1	29
	MAM	115	14	1	3
	JJA	99	13	0	14
	SON	44	20	0	11
	DJF	9	0	0	1
RGI60-13.18096	Full year	356	26	1	12
	MAM	110	3	0	2
	JJA	123	11	0	5
	SON	90	12	1	5
	DJF	33	0	0	0
RGI60-13.39432	Full year	211	9	35	30
	MAM	73	1	8	5
	JJA	60	4	2	10
	SON	63	4	21	13
	DJF	15	0	4	2
RGI60-13.49754	Full year	203	11	12	10
	MAM	68	0	5	2
	JJA	31	7	1	5
	SON	87	4	6	1
	DJF	17	0	0	2
RGI60-15.03954	Full year	276	9	26	17
	MAM	120	1	1	4
	JJA	13	4	0	3
	SON	71	1	10	4
	DJF	72	3	15	6
RGI60-15.04847	Full year	378	21	14	24
	MAM	138	3	2	4
	JJA	30	3	1	6
	SON	148	10	6	11
	DJF	62	5	5	3
RGI60-15.06065	Full year	230	16	1	9
	MAM	85	0	0	1
	JJA	12	4	0	2
	SON	80	8	1	2
	DJF	53	4	0	4

Considering full years for each glacier, the MANTRA algorithm provides a classification accuracy of 84.04 ± 6.08%. The predominant error type is overestimation (60.7%). In most cases, we found that the cause of overestimations was abundant cloud cover in the lower portions of the glacier, obscuring the actual boundary between ice and snow or casting a shadow at the boundary. Additional overestimations arise from Landsat 7’s scan line error or missing data. Underestimations occur mostly at RGI60-13.39432 and RGI60-15.03954 (61 out of 90) and are in most cases either caused by insufficient saturation in the Landsat data owing to topographic shading or assumedly by complex surface structure (e.g., on heavily fissured tongues) that may either retain snow patches or lead to strong backscattering and hence perturb the spectral signature. The number of scenes per season varies between glaciers, depending on climatic regimes, particularly seasonal distribution of cloud cover.

### Comparative analysis

Since MANTRA is the first TSLA dataset^17^ which covers HMA completely, the following cross-validation focuses on an intercomparison with two datasets from subregions: An updated version of the data by Racoviteanu *et al*.^13^ covering parts of the Hunza (Karakoram) and Langtang (Himalaya) regions while the data by Barandun *et al*.^3^ covers the Tien Shan (Fig. 4). Notably, both reference datasets are also based on (semi-)automated mapping techniques using satellite imagery. It is therefore important for the following evaluation that the reference data do not represent ‘ground truth’ but have their own uncertainties and potential error sources (Table 3). The extent of expert-driven manual corrections varies between and within the datasets as detailed below.

**Table 3:** Strengths and weaknesses of three TSLA datasets.

Dataset	Strengths	Weaknesses
RACO	Possibility of manual corrections. Highest classification accuracy (after corrections). Provides polyline of transient snowline.	Expert knowledge and manual work required. Limited potential for use in large-scale and/or long-term studies. Closed source, depends on proprietary software (ArcGIS).
SCAF	High classification accuracy. Automated, efficient algorithm. High potential for use in large-scale and/or long-term studies.	Demanding regarding in-house data storage and processing capabilities. Closed source, depends on proprietary software (IDL). Systematic errors at individual glaciers.
MANTRA	High classification accuracy. Supplementary data for each measurement. Tool to assess individual results. Highest level of automation, efficient algorithm. High potential for use in large-scale and/or long-term studies. Free, open-source software. No in-house processing capabilities required.	Depends on an external service provider (GEE). Results contain metrics only; imagery, shapes, etc. are (currently) not included.

Racoviteanu *et al*. (2018) provide snowline data for two Landsat footprints obtained by a semi-automated mapping approach combining index-based thresholding with manual corrections. The data represents subsets of the Hunza region in the Karakoram and the Langtang region in the Himalaya for the years 2013 and 2016, respectively. Each dataset consists of snowlines from nine individual Landsat-8 scenes, coded as polygons representing buffers of 100 m diameter around the snowlines. To facilitate the comparison to our TSLA data, we obtained TSLA values for individual glaciers using the same glacier outlines (RGI v6) and DEM (AW3D30) used in our approach to obtain zonal statistics of elevation (minimum, maximum, median, standard deviation) for each glacier and time step. This resulted in 1873 measurements for 472 glaciers in the Hunza and 3478 measurements for 926 glaciers in the Langtang region. The median elevation was used as a metric for the TSLA. For the intercomparison, it is important to keep in mind that here the median elevation refers to the entire snow-ice boundary which is conceptually different from the lowest two percentiles of elevation for the snow-covered area that were used in our approach. Total number of measurements coinciding with data in the MANTRA dataset amounts to 304 for the Langtang and 629 for the Hunza region.

The approach by Barandun *et al*.^3^ used an albedo-based classification approach to distinguish snow-covered and snow-free regions on glacier surfaces. They refer to their approach as Snow-Covered Area Fraction (SCAF). Landsat 5, 7 and 8 data were used as the basis for classification. Their data comprises 172,191 measurements for 1758 glaciers >2 km^2^ in the Tien Shan and Pamir for the period 2000 to 2018. The data were provided as ASCII raster files from which we obtained polygons of the snow-covered areas. Again, we applied the same glacier outlines (RGI v6) and DEM (AW3D30) used in our approach to obtain zonal statistics of elevation (minimum, maximum, median, standard deviation) for each glacier and time step. In contrast to the data by Racoviteanu *et al*. (2018), the polygons allow for application of the same TSLA metric used in our approach, i.e. the median of the lowest two percentiles of elevation for the snow-covered area. A total of 95,951 SCAF measurements could be used for intercomparison with MANTRA results.

**Fig. 5:**
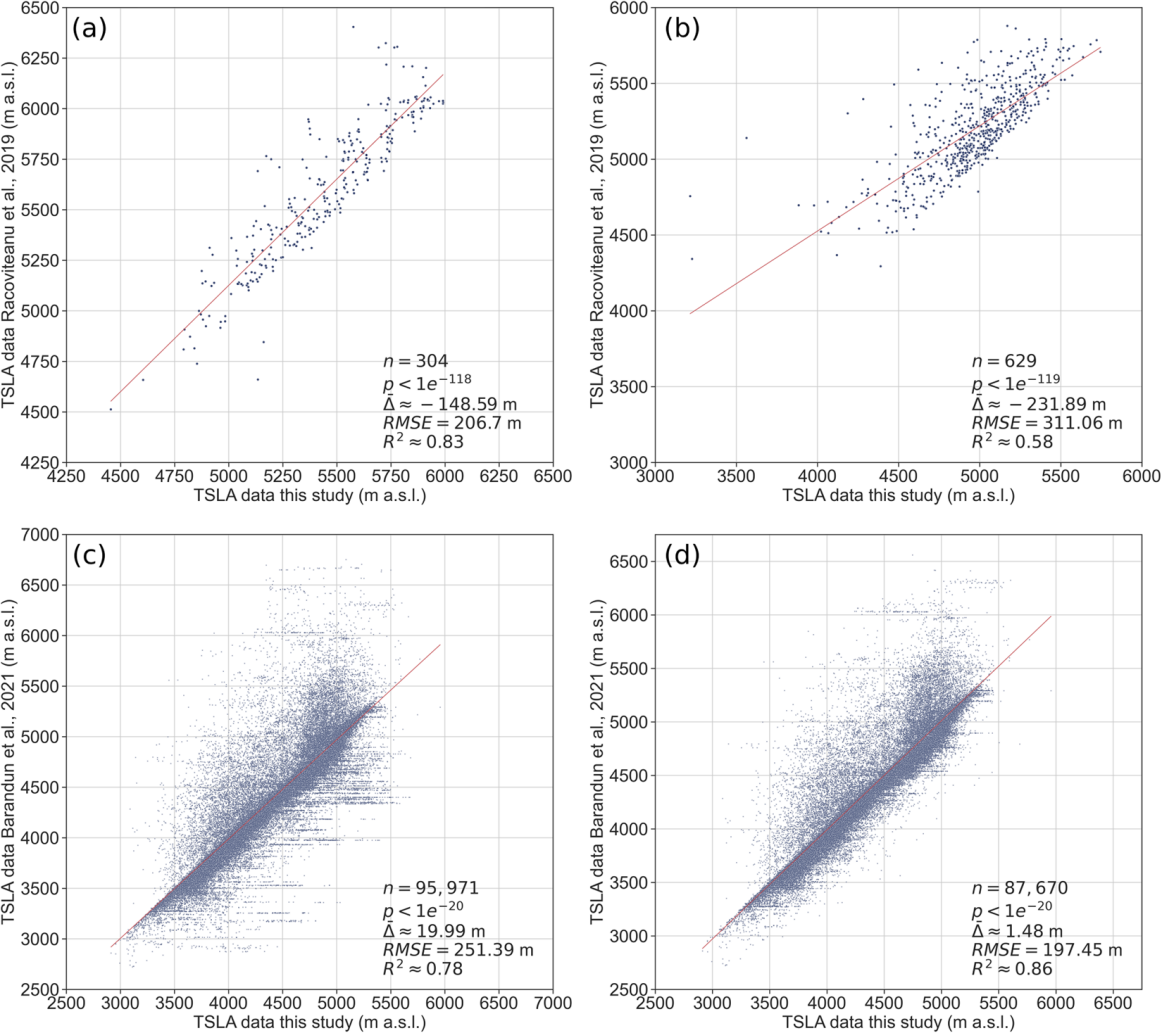
MANTRA TSLA results in comparison to data from (**a**) Racoviteanu *et al*.^13^ for the Langtang region in the Himalayas covering the year 2016, (**b**) Racoviteanu *et al*.^13^ for the Hunza region in the Karakoram covering the year 2013, (**c**) Barandun *et al*.^3^ for the Tien Shan and Pamir covering the time span 2000–2018, and (**d**) same as (**c**) but filtered for manually detected 129 glaciers with abundant (n > 3) flawed SCAF measurements.

Owing to their limited spatial and temporal coverage, data for the Langtang (Fig. 5a) and Hunza (Fig. 5b) regions comprise relatively few measurements. Negative mean deviations of −148.59 m and −231.89 m indicate that MANTRA results tend for lower TSLA estimates. Considering the different approach used by Racoviteanu *et al*.^13^ to obtain TSLA values outlined above, a systematic offset was clearly to be expected. Notably, the fit for the Langtang region is much better than for the Hunza region, as indicated by higher R² (0.83 vs. 0.58) and lower RMSE (206.7 vs. 311.06). Manual checks revealed that this is mostly due to outstandingly complex glacier geometries in the Hunza region and their effects on spectral characteristics. The most pronounced outliers are typically caused by parts of clean-ice glacier tongues being misclassified as snow in the MANTRA data, or snow misclassified as ice in the reference data.

With 19.99 m, the mean deviation between TSLAs obtained from the data by Barandun *et al*.^3^ and MANTRA data is considerably smaller (Fig. 5c). Nevertheless, the plot displays substantial scatter, evidenced by a relatively high RMSE of 251.39 m. Notably, horizontal linear clusters are clearly visible in Fig. 5c, particularly below the regression line. Manual checks revealed that many of the most pronounced deviations are caused by clear misclassifications in the SCAF dataset, i.e. high TSLA despite full snow cover or TSLA at the glacier tongue despite abundant ice. Moreover, we found that these misclassifications are not distributed arbitrarily but focus on specific glaciers. Omitting 129 glaciers with n > 3 misclassifications in the SCAF data yielded substantially improved regression metrics, with a mean deviation of 1.48 m, RMSE of 197.45 m and R² of 0.86 (Fig. 5d).

All three datasets are based on optical remote sensing data. Measurements are therefore strongly influenced by meteorological conditions and illumination. The automatic approaches of SCAF and MANTRA are subject to varying spectral characteristics of snow. Our assessment suggests that both algorithms are capable of mitigating the effects of minor changes in reflectivity. However, more pronounced deviations may cause systematic misclassifications and demand regional calibration. For example, dust deposited in accumulation areas of glaciers in arid regions of northeastern HMA causes a significant reduction in reflectivity.

## Usage Notes

The MANTRA dataset has the potential to open avenues to new research approaches in glaciology. Possible application fields include the calibration of glaciological models with temporally explicit measurement data, investigations of drivers of glacier dynamics using multivariate regression approaches, and cross-validation with other TSLA measurement data.

Using glacier outlines from RGI 6.0, the MANTRA TSLA measurements presented here consist of a single spatially integrated average (mean, median, etc.) per Landsat scene and glacier. Tributary topologies, different aspects, and other potentially relevant features of more complex glacier topographies are not considered. The current version of MANTRA offers two approaches to support further analysis of the effects of such features. Firstly, it is possible to use a manually manipulated glacier shapefile as input data e.g., sliced by tributaries or aspects. This, in theory, also applies to shapefiles of larger scales such as catchments. Secondly, geocoded raster files of spatially resolved MANTRA classification results can be downloaded from the Evaluate Tool, facilitating in-depth analyses in other geoprocessing or geoinformation environments.

Owing to the intermittent availability of suitable satellite observations, it is important to note that TSLA time series from the MANTRA dataset are gappy for individual glaciers. Chances of adverse sensing constellations during the Landsat data take, e.g. cloud cover or topographic shading in steep terrain, are varying regionally and temporally. As a consequence, quality characteristics of individual TSLA time series vary throughout the dataset, regarding total number of measurements in individual time series and the robustness of individual data points. These constraints are of limited relevance in regional assessments that integrate over (multi)annual time intervals. Before using the MANTRA data in investigations at individual glaciers, however, we strongly recommend checking the quality of the TSLA time series for the respective glacier in detail. This applies particularly when additionally focusing on individual measurements, such as TSLA maxima of specific years. Our remote sensing-based approach might not capture the exact day when the maximum TSLA was reached due to Landsat observation frequency and cloud cover at the end of the ablation phase. It is therefore important to highlight that the MANTRA dataset generally tends to underestimate annual maxima.

In this context, we would like to highlight again that annual maxima at individual glaciers will be underestimated in the respective TSLA time series in cases where no adequate Landsat imagery was available for the specific point in time when the TSL reached its highest altitude in a particular year.

Users of the MANTRA dataset for HMA should also be aware of the fact that density and spatiotemporal distribution of the measurements vary between subregions of HMA (Fig. 4). This is an inevitable consequence of meteorological drivers at the synoptic scale, including different branches of the Asian Monsoon and the Westerlies, leading to heterogeneous seasonal dynamics of cloud cover versus clear sky conditions.

Besides the relatively long time series and high density of measurements, we see the greatest prospect of the TSLA dataset in its property of being temporally and spatially explicit at the glacier scale. This opens genuine perspectives to correlate glacier dynamics to possible forcing factors, e.g., from meteorological or topoclimatic data.

## Data Availability

MANTRA is available on GitHub (https://github.com/cryotools/mantra).
